# A population-based study to investigate host genetic factors associated with hepatitis B infection and pathogenesis in the Chinese population

**DOI:** 10.1186/1471-2334-8-1

**Published:** 2008-01-02

**Authors:** Zheng Zeng, Li Guan, Ping An, Shan Sun, Stephen J O'Brien, Cheryl A Winkler, the HBV study consortium

**Affiliations:** 1 Department of Infectious Diseases, Peking University First Hospital, Beijing, P.R.China; 2 SAIC/Laboratory of Genomic Diversity, National Cancer Institute-Frederick, National Institutes of Health, Frederick, USA; 3 Conservation International (CI) China program, Beijing, P.R.China; 4 Laboratory of Genomic Diversity, National Cancer Institute-Frederick, National Institutes of Health, Frederick, USA; 5 HBV study consortium: Department of Infectious Diseases, Peking University First Hospital, Beijing, P.R.China (Zheng Zeng, Yanyan Yu, Xiaoyuan Xu, Haiying Lu); Institute of Liver Diseases Research, Beijing Military General Hospital, Beijing, P.R.China (Darong Hu); Beijing Ditan Hospital (Rongbing Wang, Yifan Chen); Department of Surgery, Beijing Institute of Tumor Prevention and Therapy, Beijing, P.R.China (Cunyi Hao); Department of Infectious Diseases, Shanxi Medical University, Taiyuan, P.R.China (Heping Zhou); Department of Infectious Diseases, Qinhuangdao No. 3 Hospital, Qinhuangdao, P.R.China (Zhonghou Han); Department of Surgery, Inner Mongolia Medical College, Hohhot, P.R.China (Lidao Bao, Xiping Zhang); Department of Infectious Diseases, Xuzhou No. 3 Hospital, Xuzhou, P.R.China (Dasi Guo); Department of Infectious Diseases, Xinjian Medical University, Urumoqi, P.R.China (Yaoxin Zhang); Department of Infectious Diseases, the Second Affiliate Hospital of China Medical University, Shenyang, P.R.China (Xiaoguang Dou); Institute of Liver Diseases Research, Peking University Second Hospital, Beijing, P.R.China (Lai Wei); Department of Surgery, Peking Union Medical College, Beijing, P.R.China (Jingan Rui, Qiang Qu)

## Abstract

**Background:**

Hepatitis B virus (HBV) infection is a significant public health problem that may lead to chronic liver disease, cirrhosis, and hepatocellular carcinoma (HCC). Approximately 30% of the world's population has been infected with HBV and approximately 350 million (5–6%) are persistent carriers. More than 120 million Chinese are infected with HBV. The role of host genetic factors and their interactions with environmental factors leading to chronic HBV infection and its complications are not well understood. We believe that a better understanding of these factors and interactions will lead to more effective diagnostic and therapeutic options.

**Methods/Design:**

This is a population-based, case-control study protocol to enroll 2200 Han Chinese from medical centers in northern and western China. Adult subjects in the following groups are being enrolled: healthy donors (n = 200), HBV infected persons achieving virus clearance (n = 400), asymptomatic HBV persistent carriers (n = 400), chronic hepatitis B cases (n = 400), decompensated liver cirrhosis with HBV infection cases (n = 400), and hepatocellular carcinoma with HBV infection cases (n = 400). In addition, for haplotype inference and quality control of sample handling and genotyping results, children of 1000 cases will be asked to provide a buccal sample for DNA extraction. With the exception of adult patients presenting with liver cirrhosis or HCC, all other cases and controls will be 40 years or older at enrollment. A questionnaire is being administered to capture dietary and environmental risk factors. Both candidate-gene and genome-wide association approaches will be used to assess the role of single genetic factors and higher order interactions with other genetic or environmental factors in HBV diseases.

**Conclusion:**

This study is designed and powered to detect single gene effects as well as gene-gene and environmental-gene interactions. The identification of allelic polymorphisms in genes involved in the pathway leading to chronic viral infection, liver cirrhosis and, ultimately, hepatocellular carcinoma would provide insights to those factors leading to HBV replication, liver inflammation, fibrosis, and the carcinogenic process. An understanding of the contribution of host genetic factors and their interactions may inform public health policy, improve diagnostics and clinical management, and provide targets for drug development.

## Background

Hepatitis B virus (HBV) infection is a significant public health problem that may lead to chronic liver disease, cirrhosis, and hepatocellular carcinoma (HCC) [1]. Approximately 30% of the world's population has been infected with HBV and approximately 350 million (5–6%) are persistent carriers. Infants infected perinatally by vertical transmission from e antigen positive mothers have a 90% risk of becoming persistent carriers. Approximately 90% of preschool children infected with HBV will fail to achieve clearance and develop persistent HBV infection. For adults, the majority of HBV-infected individuals achieve clearance with only 5–10% becoming persistent carriers of HBV. HBV accounts for 80% of all liver cancer and is an important carcinogen [2]. Of individuals persistently infected with HBV, 10–30% will develop liver cirrhosis (LC) and HCC [2]. These highly variable outcomes in both clearance rates and disease outcomes in persistently infected individuals cannot be fully explained by differences in viral or environmental factors. Thus, differences in host genetic factors may affect hepatitis B natural history.

Viral factors that may influence HBV outcomes include HBV DNA levels, HBV genotypes, HBV genetic variants, and co-infection with other hepatitis viruses. HBV DNA levels are correlated with T-cell hyporesponsiveness to HBV antigens [3] and are a risk predictor for HCC development [4,5]. Treatment with lamivudine [6] and interferon-alpha (IFN-α) [7,8] decreases viral load [3] and reduces occurrence of HCC. Of the eight HBV genotypes (A-H), HBV-A has been associated with persistence [9], HBV-C with severe liver disease [10,11], and HBV-B with more benign disease [11]; however, HBV-B was found to be a predictor for HCC [10]. A double mutation in the base core promoter of the HBV genome reported to aggravate chronic hepatitis is more frequent in HBV-C isolates than in HBV-B isolates [12]. Amino acid replacements in the "α" determinant of the HBs protein, the proposed coformational epitope essential for recognition and neutralization by anti-HBs antibodies, have been reported [13,14]. A precore stop codon mutation (1896G to A) [15] and two mutations within the core promoter region (1762A to T and 1764G to A) [16,17] have been associated with fulminant hepatitis B. Both variants show a defect in hepatitis e antigen (HBeAg) expression [18,19], which may modify the immune response of the host [20,21]. However, in many cases of fulminant hepatitis B, particularly those from nonendemic areas [22,23], neither of these mutations was observed. These investigations indicate that HBV viral burden, genotype, and genetic polymorphism are important contributors to the natural history of HBV disease and may explain, in part, the observed heterogeneity in outcomes of infection.

Environmental factors are clearly implicated in HBV pathogenesis. Alcohol and aflatoxin are two important factors that affect the progression of chronic hepatitis B. Alcohol consumption increases the severity of liver disease [24,25] and increases the risk of developing liver decompensation from cirrhosis [26]. Patients with chronic hepatitis B exposure to aflatoxins are at an increased risk of HCC [27], especially in the Fusui County of Guangxi Zhuan Autonomous Region [27,28] and the Qidong district of Jiangsu Province [29,30] in China, where the highest rates of HCC are found and aflatoxins levels are high in many local foods and grains. In Fusui County of China, the rate of HCC is 120 per 100,000 persons/year among men [31], a rate 35 times higher than that in United States. The tumor suppressor gene p53 294^ser ^mutation is a 'hotspot' mutation in HCC from patients in regions with dietary aflatoxins exposure [32,33].

The role of host genetic factors on HBV persistence and pathogenesis is less well understood. CD4+ T cell proliferative responses in acute HBV infection are significantly more vigorous than those seen in persistent HBV infection, suggesting that MHC class II polymorphisms influence susceptibility to persistent infection. Several MHC class II alleles have been identified in association with clearance or persistence of HBV infection [34-37]. Polyclonal and multispecific CD8+ T cells are readily detectable in the peripheral blood of patients with acute HBV infection or HBV clearance, but are rarely detectable in patients with persistent HBV infection. This suggests that HLA alleles may be key determinants of HBV clearance; however, there have been few comprehensive studies of HLA class I alleles and the results for class II alleles have been inconsistent [34-37]. In a comprehensive case-control study, Thio et al recently showed that the class I allele, A*301 and class II allele DRB1*1302 are associated with clearance and two class I alleles, B*08 and B*44, are associated with persistence, thus confirming an earlier studies implicating DRB1*1302 in HBV clearance [38].

Cytokines, chemokines and their receptors may also have a role in HBV persistence and disease. A number of associations with cytokines have been reported. Tumor necrosis factor (TNF)-α was associated with HBV persistence [39] and HCC [40,41]. Interleukin(IL)-10 was associated with high risk of HCC [42,43]. Genetic associations have also been observed for the mannose binding protein (MBP) [44-47] and the vitamin receptor D [48] with persistence, and hormonal markers with HCC [49,50]. Many of the genetic associations have been inconsistent among studies, possibly due to population substructure, small sample size, or differences in study design, or have not yet been replicated in duplicate studies.

These studies strongly suggest that genetic factors influence HBV disease; however, these influences are likely interactive with viral and environmental factors. The primary objective of this study is to localize and identify genes that influence HBV persistence and adverse outcomes of HBV infection by employing a population-based genetic association strategy with both candidate-gene and genome-wide association approaches. We have therefore implemented a HBV genetic study powered to detect genetic factors and their interactions in individuals representing the different stages of HBV disease: clearance, persistent infection, chronic hepatitis B, cirrhosis, and HCC, as well as normal healthy controls from the Han Chinese population in the north and west region of China (Table 1). Specific hypotheses and comparison groups are listed in Table 2.

**Table 1:** Participating hospitals or medical centers, city and province.

Hospital	City and Province
Peking University First Hospital	Beijing
Peking University Second Hospital	Beijing
Peking Ditan Hospital	Beijing
Beijing Military General Hospital	Beijing
Beijing Institute of Tumor Prevention and Therapy	Beijing
Peking Union Medical College	Beijing
Inner Mongolia Medical College	Hohhot, Inner Mongolia
Xinjiang Medical University	Urumoqi, Xinjiang Province
Qinhuangdao No. 3 Hospital	Qinhuangdao, Hebei Province
China Medical University	Shenyang, Liaoning Province
Shanxi Medical University	Taiyuan, Shanxi Province
Xuzhou No. 3 Hospital	Xuzhou, Jiangshu Province

**Table 2:** Genetic hypothesis comparison groups.

**HBV Persistence**		
**I. Persistent infection vs. clearance (B+C vs. A)**		
*Disease Category 1*	Asymptomatic HBV infection	B group
	Chromic Hepatitis B	C group
*Disease Category 2*	Clearance	A group
		
**HBV Progression**		
**II. Chronic Hepatitis B (C vs. D+E)**		
*Disease Category 1*	Chronic Hepatitis B	C group
*Disease Category 2*	Decompensated Cirrhosis	D group
	Hepatocellular carcinoma	E group
		
**III. Cirrhosis (D vs. C)**		
*Disease Category 1*	Decompensated Cirrhosis	D group
*Disease Category 2*	Chronic Hepatitis B	C group
		
**IV. Hepatocellular Carcinoma (E vs. C)**		
*Disease Category 1*	Hepatocellular carcinoma	E group
*Disease Category 2*	Chronic Hepatitis B	C group
		
**V. Asymptomatic HBV Infection (B vs. C)**		
*Disease Category 1*	Asymptomatic HBV Infection	B group
*Disease Category 2*	Chronic Hepatitis B	C group

## Methods/Design

### Recruitment sites

This study is a population-based, multicenter case-control study under a single protocol for the investigation of genetic and environmental predictors of HBV susceptibility and diseases associated with chronic HBV infection. To meet recruitment goals twelve hospitals (Table 1) are enrolling patients following protocol criteria for inclusion and exclusion. The repository, central database and coordinating center is located at Peking University First Hospital, Beijing. Blood samples are processed at local sites for isolation of peripheral blood mononuclear cells, plasma, serum and clot. All laboratory, pathology, and imaging will be done at the participating centers.

### Human subjects and enrollment criteria

#### Human subjects

Potential participants will be invited to fill in a pre-entry questionnaire for eligibility. All cases are interviewed as out- or in-patients at the participating hospitals by trained interviewers using a standard questionnaire to capture behaviors, environmental exposures, and family history related to hepatitis B infection. To reduce population heterogeneity and confounding by dietary aflatoxin exposure, only cases and controls of the Han ethnicity born and residing in north and west of China are eligible for enrollment. Local internal review board approvals from participating hospitals have been obtained and informed consent from each participant is required for study enrollment.

A total of 3200–3400 subjects will be enrolled in the study with at least 400 persons per group for HBV clearance, chronic asymptomatic HBV infection, chronic symptomatic infection, decompensated liver cirrhosis, and HCC. In addition, adult children or both parents of probands are invited to provide a buccal sample for DNA isolation; this will be used for haplotype inference and quality control of sample handling and genotyping results. Normal healthy donor controls are enrolled from local blood banks and will be negative by serological testing for HCV, HDV, and HBV. With the exception of cirrhosis and HCC cases whom are enrolled at any age of diagnosis, all cases and controls, except for those with cirrhosis or HCC, will be over 40 years of age at enrollment to provide adequate time for symptoms to manifest and to reduce probability of HBV vaccination exposure. Only probands reporting at least one parent or sibling with chronic HBV infection (HBsAg+) will be enrolled to increase the probability of HBV infection exposure at birth or early childhood.

In addition, for haplotype inference and for quality control of sample handling and genotyping results, children of 1000 cases will be asked to provide a buccal sample for DNA extraction.

#### Inclusion and exclusion criteria

The diagnostic criteria for study inclusion are listed in Table 3. Approached persons other than children or parents of probands are excluded from enrollment if they meet one or more of the exclusion criteria: 1) evidence of past or current infection by HCV or HDV; 2) birth or greater than 6 month residency in Fusui County, Guangxi Province; 3) age less than 40 for all cases and controls except HCC and cirrhosis cases; 4) other systemic disease not related to HBV infection; 5) one or more parents or grandparents not of Han ethnicity; or 6) with other hepatitis virus infection.

**Table 3:** Number of subjects and inclusion criteria

**Group A: Clearance cases (n = 400)**
1. Anti-HBs and anti-HBc positive or anti-HBs positive and no vaccination history; 2. HBV-DNA negative, HDAg negative and/or anti-HDV negative; 3. Anti HCV and HCV RNA negative; 4. ALT <40 IU/L and AST <45 IU/L at enrollment; and 5. Age ≥ 40.

**Group B: Persistent asymptomatic HBV infection (n = 400) **

1. HBsAg and anti-HBc positive; 2. Anti-HCV and HCV RNA negative; 3. Anti-HDV and/or HDAg negative; 4. At least 1 sibling or parent HBsAg positive; 5 ALT <40 and AST <45 IU/L for at least 5 years; 6. No clinical symptoms of hepatitis; 7. No clinical liver cirrhosis; and 8. Age ≥ 40.

**Group C: Chronic hepatitis B (n = 400)**

1. HBsAg and anti-HBc positive for at least 6 months; 2. Anti-HCV and HCV RNA negative; 3. Anti-HDV and/or HDAg negative; 4. At least 1 sibling or parent HBsAg positive; 5. ALT and/or AST levels greater than 2 times upper limits of normal range for testing hospital before or current; 6. No clinical evidence of liver cirrhosis; 7. Age ≥ 40.

**Group D: HBV-related decompensated liver cirrhosis (n = 400)**

1. HBsAg and anti-HBc positive; 2. Anti-HCV and HCV RNA positive; 3. Anti-HDV and/or HDAg negative; and 4. Liver cirrhosis with clinical presentation of gastroesophageal varication (3°) or a history of bleeding, or ascites, or edema, or encephalopathy, or serum Albumin < 35 g/L, total bilirubin > 35 μmol/L.

**Group E: HBV-related hepatocellular carcinoma (n = 400)**

1. HBsAg and anti-HBc positive; 2. Anti-HCV and HCV RNA positive; 3. Anti-HDV and/or HDAg negative; and 4. HCC confirmed by biopsy or elevated AFP and sonography or CT or MRI.

**Group F: Hypernormal control group (n = 200)**

1. Anti-HBs, HBsAg and anti-HBc negative and no HB vaccination history; 2. Anti-HCV and HCV RNA; 3. Anti-HDV and/or HDAg negative; 4. ALT <40 and AST <45 IU/L; and 5. Age ≥ 40.

Case definitions of HBV infection and outcomes are in accordance with the criteria issued by the Association of Infectious Diseases and Parasites Diseases of China in 2000 (Table 3) [51].

### Assay methods

#### Quantitative markers of liver function

The normal ranges for the following markers of liver function are: alanine aminotransferase (ALT) (0–40 IU/L); aspartate aminotransferase (AST) (0–45 IU/L); bilirubin (0–20 μmol/L); albumin 35–55 g/L; alpha-fetoprotein (AFP) (0–20 ng/mL).

#### Assays for hepatitis virus markers

Serum hepatitis B surface antigen (HBsAg), anti-HBs and anti-HCV antibody will be determined by the Ortho/Chemi-luminescent assay (Johnson and Johnson Co., USA). Hepatitis B e antigen and anti-HBe will be determined by the enzyme-linked immunosorbent assay using commercially available kits (AxSYM, Abbott, USA). HBV DNA will be quantified using real-time polymerase chain reaction with lower limit of detection of 1000 copies/mL. Anti-HAV IgM antibody and anti-HEV antibody will be determined by commercially ELISA kits in China. HDV antigen (HDAg) and anti-HDV antibody will also be determined by ELISA method.

### Phenotype category definitions (Table 3)

#### Clearance

Clearance is defined as a seropositive tests for antibodies against HBV surface antigen (anti-HBs) and against HBV core antigen (anti-HBc) without the presence of HBs antigen (HBsAg) at the time of study entry and to have no self-reported and/or hospital record of chronic HBV infection.

#### Asymptomatic Chronic HBV infection

Asymptomatic chronic infection is defined by two positive tests for HBsAg and antibodies to HBcAg at least 6 months apart. Alanine aminotransferase (ALT) and aspartate aminotransferase (AST) are measured by local hospital laboratories. Only individuals with normal values for ALT and AST at two timepoints separated by 12 or more months meet criteria for asymptomatic chronic infection. Laboratory measurements are obtained at study entry and from medical record review.

#### Chronic Hepatitis B

Chronic HBV infection is defined by two positive tests for HBsAg and antibodies to HBcAg at least 6 months apart and ALT and/or AST levels greater than 60 IU/L for the testing hospital before or at enrollment. To rule out confounding by co-infection with HCV or HDV, participants must be seronegative for anti-HCV, HDV antigen or anti-HDVAg, and have no detectable HCV RNA.

#### Decompensated liver cirrhosis

HBV-related decompensated liver cirrhosis is defined by liver cirrhosis in patients with persistent HBV infection and at least one of the following: 1) severe gastroesophageal varication (3°); 2) history of bleeding or current bleeding; 3) ascites or edema; 4) encephalopathy; 5) serum albumin < 35 g/L, total bilirubin >35 umol/L.

#### Primary heptocellular carcinoma

*HBV-related *HCC is defined by at least one of the following: 1) liver biopsy; or 2) elevated AFP levels and sonographic, CT, or MRI evidence.

#### Environmental risk factors

A single questionnaire is used at all enrollment sites and is administered by trained health care professionals. The instrument was developed to record self-report of risk factors for HBV transmission: family history of HBV infection, HBV-infected sexual partner(s), nosocomial exposure by invasive dental or surgical procedures, and tattooing. Past and current smoking, alcohol ingestion and exposure to dietary aflatoxin B are also captured. Demographic information will include gender, birth-date, birthplace, and past and current residency.

#### Sample size criteria

We determined that 400 each cases and controls would provide >90% power to detect moderate associations (OR ≥ 2) at α ≤ 0.05 for factors having a 10% frequency or greater (Table 4).

**Table 4:** Power calculations for the HBV study.

Allele Frequency	(n = 400 cases, 400 controls)
Odds Ratio = 1.5	% Power, alpha≤ 0.05

0.01	09
0.05	27
0.1	45
0.2	67
0.5	81

Odds Ratio = 2	

0.01	20
0.05	69
0.1	91
0.2	99
0.5	99

#### Genetic strategy

This is a population-based study that will use both candidate-gene and genome- wide association (GWA) approaches to identify genetic factors associated with clearance, persistence, cirrhosis, and HCC. A population donor control group will be used for allele frequency determination in local populations and first-degree family members will be used for haplotype determination and quality control of sample handling and genotyping. Selection of candidates will include genes encoding proteins that metabolize or transport toxins biotransformation genes, immune response genes (ligands and receptors for cytokines and chemokines, HLA, and KIR), DNA repair genes, tumor suppressor genes, genes encoding proteins used by HBV for replication, and other genes involved in the oncogenic pathways. Haplotype tagging SNPs and SNPs with known or putative function will be selected.

#### Statistics

Tests for Hardy-Weinberg equilibrium will be tested separately for cases and controls. Genotypic analyses will consider allele, dominant, recessive and additive genetic models. Haplotypes will be inferred using a Bayesian method (PHASE). Cases and controls will be group-matched and analyses will be stratified by collection site, sex, and adjusted for alcohol, smoking, diet, HBV viral levels and HBV genotype. Odds ratios will be determined using unadjusted and adjusted logistic regression analyses and the significance of association will be determined using 2-tailed Mantel-Haenszel trend test for additive models and the Chi-square or Fisher-exact test for allele, dominant and recessive genetic models.

## Discussion

Chronic HBV infection is a major contributor to morbidity and mortality worldwide despite the availability of efficient vaccines and vaccination programs in China and elsewhere [52]. The underlying environment factors contributing to HBV chronic infection are well known, but the pathophysiology of HBV-related cirrhosis and HCC are incompletely understood and the interactions between environmental and genetic factors have not been systematically explored. The susceptibility genes leading to chronic infection and subsequent disease likely affect many different pathways ranging from mediators of immune response and inflammation to oncogenic pathways. These genes may have main effects or be interactive with environmental or other genetic factors. The discovery of either main effect or interactive genes will help us to better understand HBV pathogenesis. This study is unique in that we have enrolled participants representing the spectrum of HBV exposure and pathogenesis ranging from spontaneous clearance to persistent infection leading to decompensated cirrhosis and liver cancer in a population from north and west of China (Table 1).

Heterogeneity in HBV pathogenesis and disease outcomes may be influenced by environmental factors that differ over time or geographically. In an effort to reduce bias due to changes in preventive medicine and public health measures that have impacted nutrition, HBV exposure, vaccination, and medical treatment, this study is limited to persons greater than 40 years old, except for cirrhosis and HCC. A particular problem with many genetic studies is population substructure where cases are genetically different from controls. If undetected, population substructure may lead to false discovery, even within populations considered to be genetically homogeneous [53-56]. We have attempted to minimize substructure by enrolling only Han Chinese whose primary residence is in north and west of China.

The strongest association with HCC is dietary aflatoxin exposure, a known carcinogen with high levels documented in southeast China, and particularly in Fusui County, Guangxi Province, in the Qidong district of Jiangsu Province. After 1986, food-handling practices were modified to eliminate aflatoxin contamination and food product inspections were mandated by the China government; however, aflatoxin exposure continues to be a major food contaminate in southeast China and, to lesser extent, throughout China. Limiting recruitment to hospitals in regions with lower risks for aflatoxin exposures has reduced the confounding impact of environmental dietary factors, and particularly aflatoxin exposure. Enrollment is also limited to probands and controls who are not co-infected with HCV or HDV viruses to avoid confounding by these hepatitis viruses known to interact with HBV. These inclusion and exclusion criteria are expected to increase specificity by reducing heterogeneity due to non-genetic factors.

Patients were stratified according to their infection status and the severity of their disease using stringent clinical and laboratory criteria. We anticipate that the identification of genetic risk predictors for different specific outcomes will inform clinical management of persons with chronic infection and lead to the development of better therapeutic agents. The identification of genes implicated in pathways leading to chronic HBV replication, liver inflammation, fibrosis, and the carcinogenic process will hopefully lead to improved diagnosis, risk prediction, and clinical care.

## Abbreviations

AFP: α-fetoprotein

ALT: alanine aminotransferase

AST: aspartate aminotransferase

CHB: chronic hepatitis B

CT: computed tomography

CTL: cytotoxic T lymphocyte

HBsAg: hepatitis B surface antigen

HBV: hepatitis B virus

HCC: hepatocellular carcinoma

HCV: hepatitis C virus

HDAg: hepatitis D antigen

HDV: hepatitis D virus

HLA: human leukocyte antigen

IFN: interferon

IL: interleukin

MHC: major histocompatibility complex

MRI: magnetic resonance imaging

SNP: single nucleotide polymorphism

TNF: tumor necrosis factor

TGF-β1: transforming growth factor-β1

## Competing interests

The author(s) declare that they have no competing interests.

## Authors' contributions

ZZ, CAW and SJO'B developed the protocol and study design and are responsible for the final version. PA, LG and SS contributed to the protocol and patient enrollment, respectively. CAW and ZZ wrote the manuscript.

## Pre-publication history

The pre-publication history for this paper can be accessed here:


